# Knowledge about sudden infant death syndrome prevention among postpartum women in Southern Brazil, 2019: a cross-sectional survey

**DOI:** 10.1590/S2237-96222024v33e2023622.en

**Published:** 2024-01-15

**Authors:** Anelise Medeiros Souto, Luana Patrícia Marmitt, Christian Loret de Mola, Juraci Almeida Cesar

**Affiliations:** 1 Universidade Federal do Rio Grande, Programa de Pós-Graduação em Saúde Pública, Rio Grande, RS, Brazil; 2 Universidade do Oeste de Santa Catarina, Programa de Pós-Graduação em Biociências e Saúde, Joaçaba, SC, Brazil

**Keywords:** Health Knowledge, Attitudes, Practice, Sudden Infant Death, Supine Position, Prenatal Care, Cross-Sectional Studies, Conocimientos, Actitudes y Práctica en Salud, Muerte Súbita del Lactante, Posición Supina, Atención Prenatal, Estudios Transversales, Conhecimentos, Atitudes e Prática em Saúde, Morte Súbita do Lactente, Decúbito Dorsal, Cuidado Pré-Natal, Estudos Transversais

## Abstract

**Objective:**

To assess knowledge on sudden infant death syndrome (SIDS) prevention among postpartum women who received prenatal care in public and private services in Rio Grande, Rio Grande do Sul, Brazil, in 2019.

**Methods:**

A cross-sectional study was conducted with postpartum women who gave birth in that municipality in 2019; the outcome was the indication of incorrect sleeping position (side/supine position) to prevent SIDS; the chi-square test was used to compare proportions between those who underwent prenatal care in public and private services.

**Results:**

Among all 2,195 postpartum women, 67.7% (95%CI 65.7;69.6) were unaware of the position that prevents SIDS, 71.6% were public care service users; 77.8% of them feared choking/suffocation; 1.9% were informed about SIDS during prenatal care; doctors/nurses (70.5%) and grandmothers (65.1%) were influential regarding the baby’s sleeping position.

**Conclusion:**

Most postpartum women were unaware of the sleeping position that prevents SIDS, especially those receiving care in the public sector; in general, this subject is not discussed in prenatal care.

## Study contributions

**Main results:**

Two out of three mothers believed the newborn should sleep in the side or prone position, which does not prevent but rather facilitates sudden infant death syndrome (SIDS); lack of knowledge was significantly greater when prenatal care took place in public services.

**Implications for services:**

SIDS should be addressed in prenatal care. Guidance from a doctor/nurse during consultations can be essential for mothers to change their mind and adopt a safe sleeping position (supine position) for their child.

**Perspectives:**

SIDS prevention campaigns are relevant in the context of prenatal care, as is conducting research that aims to evaluate potential impacts of interventions on the correct sleeping position for babies.

## RESUMEN

**Objetivo:**

Evaluar el conocimiento sobre la prevención del síndrome de muerte súbita del lactante (SMSL) entre puérperas que realizaron prenatal en servicios públicos y privados en Rio Grande, Rio Grande do Sul, Brasil, en 2019.

**Métodos:**

Estudio transversal, con puérperas que dieron a luz en Rio Grande, en 2019; el resultado consistió en la indicación de posición incorrecta para dormir (lado/supino) para prevenir el SMSL; utilizando chi-cuadrado, se compararon las proporciones entre mujeres que recibieron atención prenatal en servicios públicos y privados.

**Resultados:**

Entre las 2.195 puérperas, 67,7% (IC95% 65,7;69,6) desconocían como se previene el SMSL, estando el 71,6%, en la red pública; 77,8% temía asfixiarse/ahogarse; el 1,9% fue informado sobre el SMSL durante el prenatal; los médicos(as)/enfermeros(as) (70,5%) y los abuelos (65,1%) influyeron en la posición para dormir del bebé.

**Conclusión:**

La mayoría de las puérperas desconocían la posición que previene el SMSL, especialmente en la red pública; en general, este tema no está cubierto en la atención prenatal.

## RESUMO

**Objetivo:**

Avaliar o conhecimento sobre prevenção da síndrome da morte súbita do lactente (SMSL) entre puérperas com pré-natal realizado nos serviços público e privado de Rio Grande, Rio Grande do Sul, Brasil, 2019.

**Métodos:**

Estudo transversal, com puérperas do município; seu desfecho constituiuse da indicação de posição incorreta para dormir (decúbito lateral ou dorsal), visando prevenir a SMSL; utilizou-se o teste qui-quadrado para comparar proporções do desfecho e de exposição entre puérperas que realizaram pré-natal nos serviços público e privado.

**Resultados:**

De 2.195 puérperas, 67,7% (IC_95_% 65,7;69,6), majoritariamente atendidas na rede pública (71,6%), desconheciam a posição preventiva da SMSL; 77,8% temiam engasgo/afogamento; 1,9% foram informadas sobre SMSL no pré-natal; médicos(as)/enfermeiros(as) (70,5%) e avós (65,1%) mostraram-se influentes na decisão sobre como posicionar o bebê adormecido.

**Conclusão:**

A maioria das puérperas, especialmente as atendidas na rede pública, desconhecia a posição que previne SMSL; geralmente, o tema não é abordado no pré-natal.

## INTRODUCTION

Sudden Infant Death Syndrome (SIDS) is the name given to the unexpected, incomprehensible occurrence, without apparent reason, of a death of a child under 1 year old, even after assessment of the scene of death, clinical history and autopsy.^1,2^ Although the cause is unknown, it is believed that SIDS results from difficulty in the infant regulating heartbeat, breathing and body temperature, especially during sleep.^3^

The position in which the child sleeps is decisive for the occurrence of this type of death. Sleeping in the supine position (face or belly up) prevents SIDS, while sleeping in the prone position (face or belly down) or lateral position (on its side) favors its occurrence. Practices such as bed sharing (sleeping in the same bed as its parents), using a very soft mattress and/or excessive covering contribute to increasing the risk of SIDS.^4^

Prenatal care aims to prevent, diagnose, treat and adequately manage a range of diseases and unfavorable situations, aiming to ensure that there are no complications during pregnancy, childbirth and the postpartum period.^5^ A variety of information is provided and care is offered to pregnant women during prenatal consultations. Although prenatal coverage has increased worldwide, SIDS is the third leading cause of infant death among all countries and the leading cause in developed countries.^6^

In the United States, it is estimated that SIDS accounts for around 2,000 deaths annually.^7^ In developing countries, such as Brazil, SIDS incidence is frequently underreported, given (i) the difficulty in establishing an accurate diagnosis of the event and (ii) the absence of mandatory necropsy, making it difficult to record deaths due to this cause.^8,9^ In 2021, 173 deaths attributed to SIDS were reported on the Health Ministry Mortality Information System (*Sistema de Informações sobre Mortalidade* - SIM);^10^ in the same year, 957 deaths of children in the first year of life were recorded as having an “ill-defined” cause, suggesting the possibility that SIDS, as an underlying cause of child deaths, was underreported. 

SIDS is however easy to prevent. Sleeping in the supine position is enough to prevent its occurrence in up to 70% of cases.^4,9^ This preventive approach to SIDS, however, is not common practice in the training of health professionals.^11,12^ Furthermore, there is no information available, in population-based studies, that this subject is addressed with pregnant women during prenatal consultations; if it is not addressed, from whom they learn the position in which to put their baby to sleep safely; and whether this knowledge differs between those who received prenatal care in public services and those who received it in private services.^13^

The objective of this study was to assess knowledge about prevention of SIDS among postpartum women who underwent prenatal care in public and private health services in the city of Rio Grande, state of Rio Grande do Sul, Brazil, in 2019.

## METHODS

This was a cross-sectional study, conducted in Rio Grande, on the south coast of the state of Rio Grande do Sul, 300 km from the state capital Porto Alegre. At the time, 2019, the municipality had 212,000 inhabitants and its municipal human development index was 0.744; while the infant mortality rate was 11.9 per 1,000 live births,^9^ this being higher than the state average of 10.6 per 1,000 live births.^14^ The study included all postpartum women who gave birth between January 1^st^ and December 31^st^, 2019, in Rio Grande’s two maternity hospitals: *Santa Casa de Misericórdia de Rio Grande*; and *Hospital Universitário Dr. Miguel Riet Corrêa Jr*., linked to the *Universidade Federal do Rio Grande* (FURG). Postpartum women who lived in the urban or rural area of the municipality and had attended at least one prenatal consultation, whose child’s birth weight was equal to or greater than 500 grams or was born at gestational age of at least 20 weeks were eligible.

Data relating to the demographic and reproductive characteristics, lifestyle habits and behavior of those mothers, in addition to their knowledge about preventing sudden infant death, were collected by means of a questionnaire administered by three interviewers, within a period of up to 48 hours after birth, when they were still were in hospital. Tablets and the REDCap (Research Electronic Data Capture)^15^ application were used to collect the data, with daily download to the FURG central server. Details about the methodology applied are available in a previous publication.^16^

The outcome under study was verified based on the following question:

*In which position do you think a baby should sleep? (1) Face down; (2) face up; (3) on its side; (4) other answer, which was written down and later coded; and (9) I don’t know.*

All postpartum women whose answer was not option (2), sleeping on its back (supine position), were considered as being unaware of the safest sleeping position for a newborn – a positive answer for the study outcome. 

The questionnaire was designed so that the variables to be studied formed blocks of information distributed as follows:

a) Demographic and socioeconomic variables
  - maternal age (in years);
  - race/skin color (White; mixed race; Black);
  - number of dwellers in the household (2-3; 4-5; 6 or more);
  - lived with a partner (yes; no);
  - schooling (in years: 0-4; 5-8; 9-11; 12 or more);
  - paid work during pregnancy (yes; no);
  - partner unemployed (yes; no); and
  - family income (in BRL, for the month immediately prior to the interview), taking all dwellers in the household.
b) Reproductive variables
  - primiparous (yes; no); and
  - prior miscarriage (yes; no).
c) Lifestyle habit variables
  - smoking before and during pregnancy (yes; no);
  - gestational age when prenatal consultations began;
  - number of prenatal consultations; and
  - care received during pregnancy and childbirth.

Based on this information, each woman was classified as having had adequate prenatal care when:

- they began prenatal consultations in the first trimester;
- had six or more consultations; and
- tested at least twice for HIV, syphilis and qualitative urine analysis.

The final block of the questionnaire dealt with care at childbirth, that is: whether delivery was performed by a doctor, whether the woman was attended to by a doctor; whether the woman in labor underwent episiotomy; whether she was cared for in Brazilian National Health Service (Sistema Único de Saúde - SUS) facilities (yes; no); and whether childbirth was vaginal or cesarean. 

Regarding sleeping position, questions were asked on the reasons for putting their baby in the position they reported and from whom the mother obtained this information, with special emphasis on whether this happened:

a) during prenatal consultations;
b) due to guidance from a family member; or
c) because of the *Pastoral da Criança* “Sleeping face up is safer” national campaign.

The postpartum women were then asked:

a) whether they would accept putting their baby to sleep in the supine position (face/belly up) if this was recommended by a health professional, especially a doctor and/or a nurse;
b) whether they believed that sleeping in the supine position can avoid sudden death; and
c) whether they intended to put their baby to sleep in the supine position.

Around 10% of the interviewers’ questions were asked again by one of the study supervisors, within 15 days of hospital discharge. The objective of this procedure was to confirm that the questionnaire had been administered and to assess the degree of agreement between the answers, whereby the Cohen’s Kappa index ranged from 0.61 to 0.99: the vast majority of interviewees were found to have agreement above 0.72 – considered to be “very satisfactory”.^17^

The sample size calculation was carried out *a posteriori*, since the data had already been collected. Considering the available “n” of 2,195 postpartum women, 67.7% prevalence of the outcome and a 95% confidence level, the margin of error of the estimates presented is 2.0 percentage points at the most.^16^

The initial analysis consisted of checking inconsistent values, categorizing derived variables and comparing proportions, using Pearson’s chi-square test and Fisher’s exact test; Student’s *t* test was applied to compare averages. Interaction was then assessed and the variables were then stratified according to the nature – public or private – of prenatal care: those who had the majority of their consultations via the SUS were characterized as having had prenatal care in public services, while the other postpartum women, who paid for this service, in full or in part, directly or through health insurance, were characterized as having received prenatal care in private services. The Stata 11.0 statistical package was used for all analyses; a 95% statistical significance level was adopted. 

The research project was submitted to the *Universidade Federal do Rio Grande* Health Research Ethics Committee, and was approved as per Opinion No. 278/2018. All participants signed a Free and Informed Consent Form (TCLE), given that the confidentiality of answers, voluntary participation and the possibility of leaving the study at any time were guaranteed.

## RESULTS

Of the 2,317 postpartum women who gave birth in the two maternity hospitals in Rio Grande in 2019, 2,270 (98.0%) were interviewed; of these, 75 (3.3%) were excluded from the study because they had not received prenatal care, leaving 2,195 who had had at least one prenatal consultation during that period. Tables 1, 2 and 3 compare the prevalence of several indicators, between postpartum women who received prenatal care in public health services and those cared for in private health services. 

In Table 1, it can be seen that those cared for in public services were, on average, 3.3 years younger, had a higher proportion of mixed and Black race/skin color, reported a greater number of household dwellers, had a partner less frequently, had, on average, four years less schooling and, to a lesser extent, carried out paid work during pregnancy; however, the proportion of women cared for in public health services who smoked during pregnancy was 6.8 times higher than the same proportion among postpartum women cared for in private services. Regarding prenatal care, the proportion of postpartum women cared for in public services who started consultations in the first trimester, had six or more consultations and completed prenatal care considered adequate was lower, when compared to postpartum women cared for in private services. 

**Table 1:** Demographic, socioeconomic and reproductive characteristics and lifestyle habits of postpartum women who had children (n = 2,195), according to the nature of the prenatal care service, Rio Grande, Rio Grande do Sul, Brazil, 2019

Characteristics/lifestyle habits	Total			Prenatal care in public services			Prenatal care in private services		p-value^b^
	**N**	**%**		**n**	**% (95%CI^a^)**		**n**	**% (95%CI ^a^ )**	
Age (in years)									< 0.001
11-19	285	13.0		255	18.3 (16.3;20.4)		30	3.8 (2.6;5.3)	
20-24	601	27.4		427	30.6 (28.2;33.1)		174	21.8 (19.0;24.7)	
25-29	514	23.4		301	21.6 (19.5;23.8)		213	26.5 (23.7;29.8)	
30-47	795	36.2		412	29.5 (27.2;32.0)		383	47.9 (44.4;51.3)	
**Race/skin color**									**< 0.001**
White	1,689	77.0		1,000	71.7 (0.69;74.0)		689	86.2 (83.5;88.3)	
Mixed race	334	15.2		254	18.2 (16.3;20.3)		80	10.0 (8.1;12.3)	
Black	172	7.8		141	10.1 (8.6;11.8)		31	3.8 (2.7;5.4)	
**Dwellers in household**									**< 0.001^c^**
2-3	1,365	62.2		758	54.4 (51.7;56.9)		607	75.9 (72.8;78.7)	
4-5	683	31.1		504	36.1 (33.6;38.7)		179	22.4 (19.6;25.4)	
≥ 6	147	6.7		133	9.5 (8.1;11.2)		14	1.7 (1.0;2.9)	
**Lived with a partner**	**1,884**	**85.8**		**1,139**	**81.6 (79.6;83.7)**		**745**	**93.1 (91.4;0.95)**	**< 0.001**
**Schooling (in years of study)**									
≤ 4	80	3.6		72	5.2 (4.1;6.5)		8	1.0 (0.5;2.0)	< 0.001^c^
5-8	583	26.6		533	38.1 (35.6;40.7)		51	6.4 (4.9;8.3)	
9-11	1,044	47.6		660	47.3 (44.7;50.0)		384	48.0 (44.5;51.5)	
≥ 12	488	22.2		131	9.4 (8.0;11.0)		357	44.6 (41.2;48.1)	
**Paid work during pregnancy**	**951**	**43.3**		**432**	**31.0 (28.5;33.4)**		**519**	**64.9 (61.6;68.2)**	**< 0.001**
**Partner unemployed**	**308**	**14.7**		**240**	**18.4 (16.4;20.6)**		**68**	**8.6 (6.8;10.8)**	**< 0.001**
**Monthly family income (in minimum wages^d^)**									**< 0.001^c^**
≤ 0.9	193	9.0		183	13.6 (11.8;15.5)		10	1.3 (0.7;2.3)	
1.0-1.9	733	34.2		632	46.9 (44.3;49.6)		101	12.7 (10.6;15.2)	
2.0-3.9	873	40.8		476	35.3 (32.8;37.9)		397	50.0 (46.5;53.5)	
≥ 4	342	16.0		56	4.2 (3.2;5.4)		286	36.0 (32.7;39.4)	
**Primiparous**	**846**	**38.5**		**483**	**34.6 (32.1;37.2)**		**363**	**45.4 (41.9;48.8)**	**< 0.001**
**Prior miscarriage**	**342**	**15.6**		**212**	**15.2 (13.4;17.2)**		**130**	**16.3 (13.8;19.0)**	**0.510**
**Smoking before and during pregnancy**	**248**	**11.3**		**229**	**16.4 (14.6;18.5)**		**19**	**2.4 (1.5;3.7)**	**< 0.001^c^**
**Consultations begun in first trimester of pregnancy**	**1,789**	**81.5**		**1,062**	**76.1 (73.8;78.3)**		**727**	**90.9 (89.7;92.7)**	**< 0.001**
**Six or more consultations**	**1,948**	**88.7**		**1,191**	**85.4 (83.4;87.1)**		**757**	**94.6 (92.8;96.0)**	**< 0.001**
**Adequate prenatal care^e^**	**1,453**	**66.2**		**863**	**61.9 (59.3;64.4)**		**590**	**73.8 (70.6;76.7)**	**< 0.001**
**TOTAL**	**2,195**	**100.0**		**1,395**	**63.5 (61.5;65.5)**		**800**	**36.5 (34.5;38.5)**	

a) 95%CI: 95% confidence interval; b) Pearson’s chi-square test; c) Fisher’s exact test; d) The minimum wage was taken to be BRL 998; e) Adequate prenatal care was taken to be six or more consultations, starting in the first trimester of pregnancy, performance of two or more qualitative urine tests and two or more diagnostic tests for human immunodeficiency virus (HIV) and syphilis.

As shown in Table 2, 65.4% of mothers stated that newborn babies should sleep in the side or prone position, with this proportion being higher among those who received prenatal care in public services (71.6%), compared to those cared for in the private sector (54.6%). Among all the postpartum women interviewed, the main reason for this choice was to prevent the child from suffocating/choking – regardless of whether prenatal care was provided through public or private services; or regardless of the recommended sleeping position for newborns. Still in relation to all the interviewees, whether cared for in public or private healthcare, around half stated that they learned about the correct position by themselves. Among those who stated that the supine sleeping position was the safest for newborns, 17.4% said they had gained this knowledge through the SIDS prevention campaign run by the *Pastoral da Criança*, while 14.1% said they had received guidance from doctors and/or nurses: 13.5% (95%CI 10.3;17.4) of postpartum women whose prenatal care was provided by the public sector; and 14.8% (95%CI 11.4;19.1) of those who had prenatal care in the private sector. We also found that only 1.9% of all postpartum women reported the topic being addressed in a prenatal consultation, with no difference in terms of public or private services.

**Table 2:** Distribution of the postpartum women (n = 2,195) regarding their opinion on the position in which the newborn should sleep, according to the nature of the prenatal care service, Rio Grande, Rio Grande do Sul, Brazil, 2019

Postpartum women’s knowledge/opinion	Total			Prenatal care in public services			Prenatal care in private services		p-value^b^
	**N**	**%**		**n**	**% (95%CI^a^)**		**n**	**% (95%CI^a^)**	
**Position in which the newborn should sleep**									**< 0.001^c^**
Supine (face up)	708	32.3		364	26.1 (23.8;28.5)		344	43.0 (39.6;46.5)	
Lateral (on its side)	1,433	65.3		997	71.5 (69.0;73.8)		436	54.5 (51.0;57.9)	
Prone (face down)	3	0.1		2	0.1 (0.01;0.1)		1	0.1 (0.01;0.8)	
Does not know	51	2.3		32	2.3 (1.6;3.2)		19	2.4 (2.5;3.7)	
**Reasons for choosing any of these positions**									**< 0.001^c^**
Avoid choking/suffocation	1,695	77.8		1,091	78.9 (76.7;81.0)		604	75.8 (72.7;78.6)	
Provide greater comfort	340	15.6		222	16.1 (14.2;18.1)		118	14.8 (12.5;17.4)	
Health professional guidance	38	1.8		15	1.1 (0.06;1.8)		23	2.9 (1.9;4.3)	
Positive previous experience	16	0.7		11	0.8 (0.04;1.4)		5	0.6 (0.03;1.5)	
Knowledge gained through national campaign	14	0.6		3	0.2 (0.07;6.7)		11	1.4 (0.08;2.5)	
Maternal grandmother’s guidance	11	0.5		7	0.5 (0.02;1.6)		4	0.5 (0.02;1.3)	
Other	65	3.0		33	2.4 (1.7;3.3)		32	4.0 (2.8;5.6)	
**Reasons for choosing the supine position (face up)^d^ (n = 708)**									**< 0.001**
Avoid choking/suffocation	385	54.4		179	49.2 (44.0;54.3)		206	59.9 (54.6;65.0)	
Provide greater comfort	241	34.0		152	41.8 (36.8;46.9)		89	25.9 (21.5;30.8)	
Other	82	11.6		33	9.0 (6.5;12.5)		49	14.2 (10.9;18.4)	
**Reasons for choosing the side position^e^**									**0.603^c^**
Avoid choking/suffocation	1,307	91.3		911	91.4 (89.5;93.0)		396	90.8 (87.7;93.2)	
Provide greater comfort	93	6.5		66	6.6 (5.2;8.3)		27	6.2 (4.3;8.9)	
Other	33	2.2		20	2.0 (1.2;3.0)		13	3.0 (1.7;5.1)	
**Who taught you the put the newborn to sleep in the supine position^d^**									**0.019**
Newborn’s maternal grandmother	47	6.6		25	6.9 (4.7;10.0)		22	6.4 (4.2;9.5)	
No one/by yourself	376	53.1		209	57.4 (52.2;62.4)		167	48.6 (43.3;53.8)	
Doctor/nurse	100	14.1		49	13.5 (10.3;17.4)		51	14.8 (11.4;19.1)	
*Pastoral da Criança* Campaign	123	17.4		47	12.9 (9.8;16.8)		76	22.1 (18.0;26.8)	
Other	62	8.8		34	9.3 (6.7;12.8)		28	8.1 (5.7;11.6)	
**Who taught you the put the newborn to sleep in the side position**									**0.208^c^**
Newborn’s maternal grandmother	686	47.9		486	48.8 (45.6;51.9)		200	45.9 (41.2;50.6)	
No one/by yourself	720	50.2		493	49.5 (46.3;52.6)		227	52.1 (47.4;56.7)	
Doctor/nurse	4	0.3		1	0.1 (0.01;0.7)		3	0.7 (0.2;2.1)	
*Pastoral da Criança* Campaign	2	0.1		1	0.1 (0.01;0.7)		1	0.2 (0.03;1.6)	
Other	21	1.5		16	0.5 (0.1;2.6)		5	1.1 (0.5;2.7)	
**Guidance during prenatal consultations on the safest sleeping position for the newborn**									**0.470^c^**
	42	1.9		26	1.9 (18.6;27.2)		16	2.0 (1.2;3.2)	

a) 95%CI: 95% confidence interval; b) Pearson’s chi-square test; c) Fisher’s exact test; d) n = 708; e) n = 1,433.

In Table 3, it can be seen that the most influential people in the maternal decision to put the newborn to sleep in the supine position were doctors and/or nurses (70.5%), followed by the newborn’s maternal grandmother (65.1%), in the two prenatal service locations in Rio Grande. Just over half of all of them (52.4%) believed that putting newborns to sleep in the supine position would prevent SIDS, with this percentage being significantly higher among those who received prenatal care in the private sector (63.5%; 95%CI 60.1;66.8) compared to those cared for in the public sector (46.0%; 95%CI 43.4;48.6). One in every six postpartum women (16.5%) said they were aware of the National Campaign run by the *Pastoral da Criança*; this proportion was two times higher among those who received prenatal care in the private sector (25.6%; 95%CI 22.7;28.8), compared to those who received it in the public sector (11.2%; 95%CI 9.6;12.9). Also with regard to the private sector, there was a higher proportion of postpartum women who intended to put their newborn to sleep in the supine position (57.3%; 95%CI 53.8;60.7) (Table 3).

**Table 3:** Opinion of postpartum women (n = 2,195) on situations related to preventing sudden infant death , according to the nature of the prenatal care service, Rio Grande, Rio Grande do Sul, Brazil, 2019

Situations	Total			Prenatal care in public services			Prenatal care in private services		p-value^b^
	**N**	**%**		**n**	**% (95%CI^a^)**		**n**	**% (95%CI^a^)**	
Would accept putting newborn to sleep face up if so recommended by									
Doctor/nurse	1,547	70.5		928	66.6 (64.0;69.0)		619	77.4 (74.3;80.1)	< 0.001
Newborn’s maternal grandmother	1,428	65.1		858	61.5 (58.9;64.0)		570	71.1 (68.0;74.3)	< 0.001
**Believes that sleeping in the supine position can avoid death of newborn**									
	1,150	52.4		642	46.0 (43.4;48.6)		508	63.5 (60.1;66.8)	< 0.001
**Has seen the** *Pastoral* *da* *Criança* **“Sleeping face up is safer” campaign**									
	361	16.5		156	11.2 (9.6;12.9)		205	25.6 (22.7;28.8)	< 0.001
**Intends to put the newborn to sleep in the supine position**									
	1,048	48.0		591	42.7 (40.1;45.3)		457	57.3 (53.8;60.7)	< 0.001

a) 95%CI: 95% confidence interval; b) Pearson’s chi-square test.

## DISCUSSION

The results of this study revealed that for two out of three postpartum women, the newborn should sleep in the lateral or prone position, a position that does not prevent, but rather facilitates the occurrence of SIDS. This proportion was significantly higher among those who received prenatal care in public health services. It was also found that the position chosen as “the safest” aims much more to prevent the infant from suffocating/choking than to actually prevent SIDS. Only 1.9% of all postpartum women reported that the topic was discussed in a prenatal consultation, which led them to learn, by themselves, the correct position for the sleeping child. Finally, the enormous potential that doctors, nurses and maternal grandmothers have to change the mother’s opinion about the newborn’s sleeping position stood out; as did the fact that mothers care for in Rio Grande public health services received poorer quality prenatal care.

The vast majority of mothers – around 68% – indicated positions other than the safest sleeping position (supine or belly-up position) for the newborn, with a view to preventing SIDS. This lack of knowledge was even greater among pregnant women whose prenatal care was provided in public services, approximately 72% of them. In fact, the positions mentioned by most mothers (side position mainly; prone position sometimes) end up favoring sudden death, rather than preventing its occurrence. This prevalence of inadequate position, reported by Rio Grande mothers, is lower than the 80% prevalence found in 2016 (three years earlier) for the same municipality of Rio Grande,^13^ and the 78% prevalence found in Passo Fundo, another municipality in the state of Rio Grande do Sul, in 2004,^8^ although the finding of the present study is still higher than the 45% prevalence reported in 2015, for Pelotas, a municipality neighboring Rio Grande.^18^

Although the rate of lack of knowledge about the safe position for infants to sleep in has shown a downward trend among Rio Grande women – from 80% to approximately 68%, in three years –, the proportion of mothers in the municipality who are unaware of the supine position as the only one capable of preventing SIDS remains high.^13^ This finding indicates that there is still a long way to go before all pregnant and postpartum women become informed about SIDS and safe sleeping positions. In high-income nations, such as the Netherlands, the United Kingdom, New Zealand, Australia, Scandinavian countries and the United States, where campaigns to publicize the supine position have been put in place, the rate of children sleeping in the prone or side position has decreased by between 50% and 90%, and the SIDS rate has fallen in the same proportion.^19^ This simple measure, implemented over the last three decades, has been attributed to the reduction of around 3,000 infant deaths in New Zealand, 17,000 in England and Wales, and approximately 40,000 in the United States.^20^ On the other hand, Brazil has had just one national campaign, run by the *Pastoral da Criança* in 2009. 

Mothers in Rio Grande were much more concerned about the possibility of their child suffocating/choking than about sudden death. For this reason, two out of three of them demonstrated the intention of putting their baby to sleep on its side, an incorrect choice because it is unsafe: the newborn may move from this position, to the point of having restricted breathing, especially if there are blankets, pillows or other soft objects around it.^21^ This is particularly serious because at this age the brain, although immature, is incapable of recognizing situations of respiratory difficulty, suffocation or even apnea, and by not reacting to them, the child can die suddenly and silently.^7,18^ After all, any sleeping position other than the supine position favors the occurrence of sudden infant death. Therefore, it is essential that mothers adopt safe sleeping practices, such as putting their baby to sleep lying on its back, with the environment within its reach free of soft objects; and just as important, not sharing a bed with it.^4^

The lack of knowledge and inadequate case management found in this study show that the subject is not addressed in routine prenatal consultations. However, it is noteworthy that 70% of mothers mentioned that, when putting their child to sleep, they would adopt the supine position if it were recommended by a doctor or nurse. In fact, prevalence of safe infant sleeping practices increased by 28% among mothers correctly informed by a health professional.^22^

Lack of knowledge about the safest sleeping position is further aggravated when one notes that correct guidance is more common among women who receive prenatal care in private services, compared to those receiving care in public services. In Pelotas, for example, while 76% of the mothers cared for were recommended to adopt the safest sleeping position for their newborns, only 48% received this guidance in public services.^18^ This is very serious, because it is precisely the women cared for by public health services who present a combination of factors that are more favorable to the occurrence of SIDS.^10^

In practically all aspects evaluated, mothers who received prenatal care via the SUS were at a clear disadvantage compared to the others who were cared for in the private sector, which has been repeatedly reported for a long time in the so-called Southern Half of the state of Rio Grande do Sul.^23^ A similar pattern has been found in relation to knowledge about SIDS. Therefore, all actions aimed at improving prenatal care and preventing the occurrence of SIDS must prioritize mothers cared for by Brazilian National Health System services, aiming to further reduce this health inequity in the region. 

Almost half of the mothers intended to put their baby to sleep lying on its side based on maternal grandmother guidance, which demonstrates the strong influence of this family character on a mother’s decision, which is of vital importance for her baby. A study conducted in the United States demonstrated that, when sleeping at its grandmother’s house, 58% of the time, the baby was placed in the supine position, compared to 45% in the situation in which the grandmother put it to sleep at her daughter’s home.^24^ The United States study also pointed out that, for grandmothers, the supine position increased the risk of suffocation, at the same time that it seemed more comfortable for the newborn to be placed in a lateral or prone position, above all.^24^ It is clear that when dealing with this issue campaigns must necessarily include grandmothers, otherwise their impact will be far less than desired.

When interpreting the results of this study, some inherent limitations must be taken into account. Firstly, we sought to investigate the mother’s knowledge and intention of putting her child to sleep in a given position, and not necessarily whether she really will do so; that is, it is not certain whether, in fact, the mother will act in this way. It is quite possible that, due to the influence of maternal grandmothers, who were largely in favor of putting newborns to sleep in the supine or side position, the proportion of newborns sleeping in an unsafe position may be even higher. It is also worth highlighting the fact that 75 postpartum women (3.3% of the initial study sample) did not have a single prenatal consultation and were therefore excluded from the analysis, because the main objective of the authors was to compare indicators on postpartum women who received prenatal care in the public sector *versus* postpartum women who received care in the private sector. Despite not having received prenatal care, the question about the sleeping position was nevertheless asked: 81.3% (61 of these 75 mothers) answered that newborns should sleep in a lateral or supine position. This demonstrates that the exclusion of these mothers led to underestimation of the outcome since, among all interviewees, reported prevalence of newborns in unsafe sleeping positions was 67.7%; if they were included, prevalence would increase to 70.4%, that is, 4.1% higher, which makes the results of this study even more relevant and worrying. In terms of the virtues of this study, it is worth highlighting the high response rate, 98% – this being more than representative, census-like – for a medium-sized municipality such as Rio Grande.

The findings of this research not only demonstrated that few mothers knew the correct way to prevent SIDS. They also revealed that the topic is not discussed as part of the routine of prenatal consultations, which is why mothers take the initiative to seek this knowledge by themselves, turning mainly to the baby’s maternal grandmother. It is also clear how much guidance from a doctor or nurse was mentioned by them as being essential for changing their opinion and adopting a safe sleeping position for their babies.

Health service managers are advised of the need to run a campaign that recommends the correct and safest newborn sleeping position to mothers – and maternal grandmothers – i.e. the supine position. This could be done by making a note in the Pregnant Women and Children’s Health Card, or through the use of posters and folders; and even national mass campaigns. It is recommended that health professionals approach the subject during prenatal consultations, not only because of the practically zero cost for health services but, mainly, because of the enormous impact that recommendations made by health professionals would have in reducing mortality to up to 3 deaths per 1,000 live births, which would not be a small reduction considering that the current rate continues to be around 10/1,000 live births in the municipality. 

Finally, for researchers interested in and dedicated to the topic, we recommend developing and conducting studies on how much the mothers’ expressed intention actually translates into the action they report, as well as studies evaluating the potential impact of an educational intervention on the safest sleeping position for newborns, in order to reduce the occurrence of SIDS in the municipality of Rio Grande. 
